# FGF15 promotes hepatic NPC1L1 degradation in lithogenic diet-fed mice

**DOI:** 10.1186/s12944-022-01709-8

**Published:** 2022-10-08

**Authors:** Pingfan Mo, Hongtan Chen, Xin Jiang, Fengling Hu, Fenming Zhang, Guodong Shan, Wenguo Chen, Sha Li, Yiqiao Li, Guoqiang Xu

**Affiliations:** 1 Department of Gastroenterology, Zhejiang University School of Medicine, The First Affiliated Hospital, 79 Qingchun Road, Hangzhou, 310006 Zhejiang China; 2 Urology& Nephrology Center, Department of Nephrology, Zhejiang Provincial People’s Hospital and Hangzhou Medical College Affiliated People’s Hospital, 158 Shangtang Road, Hangzhou, 310014 Zhejiang China

**Keywords:** Cholesterol gallstone disease, NPC1L1, Ezetimibe, Ubiquitination, FGF15

## Abstract

**Background:**

Cholesterol gallstone disease (CGD) is accompanied by biliary cholesterol supersaturation. Hepatic Niemann-Pick C1-like 1 (NPC1L1), which is present in humans but not in wild-type (WT) mice, promotes hepatocyte cholesterol uptake and decreases biliary cholesterol supersaturation. In contrast, intestinal NPC1L1 promotes intestinal cholesterol absorption, increasing biliary cholesterol supersaturation. Ezetimibe (EZE) can inhibit both hepatic and intestinal NPC1L1. However, whether hepatic NPC1L1 can affect CGD progress remains unknown.

**Methods:**

Mice expressing hepatic NPC1L1 (NPC1L1^hepatic-OE^ mice) were generated using Adeno-associated viruses (AAV) gene delivery. The protein level and function of hepatic NPC1L1 were examined under chow diet, high fat-cholesterol diet (HFCD), and lithogenic diet (LD) feeding. Gallstone formation rates were examined with or without EZE treatment. Fibroblast growth factor 15 (FGF15) treatment and inhibition of fibroblast growth factor receptor 4 (FGFR4) were applied to verify the mechanism of hepatic NPC1L1 degradation.

**Results:**

The HFCD-fed NPC1L1^hepatic-OE^ mice retained the biliary cholesterol desaturation function of hepatic NPC1L1, whereas EZE treatment decreased biliary cholesterol saturation and did not cause CGD. The ubiquitination and degradation of hepatic NPC1L1 were discovered in LD-fed NPC1L1^hepatic-OE^ mice. Treatment of FGF15 during HFCD feeding and inhibition of FGFR4 during LD feeding could affect the protein level and function of hepatic NPC1L1.

**Conclusions:**

LD induces the ubiquitination and degradation of hepatic NPC1L1 via the FGF15-FGFR4 pathway. EZE may act as an effective preventative agent for CGD.

**Supplementary Information:**

The online version contains supplementary material available at 10.1186/s12944-022-01709-8.

## Introduction

Cholesterol gallstone disease (CGD) is a common digestive system disease [1] that is usually accompanied by hypercholesterolemia (LDL-cholesterol) [2] and biliary cholesterol supersaturation caused by the increased dietary cholesterol absorption in the ileum [3]. Niemann-Pick C1-like 1 (NPC1L1) is a polytopic transmembrane protein that contain a sterol-sensing domain, essential to promote the ability to absorb cholesterol [4, 5]. As an inhibitor of NPC1L1 [6], ezetimibe (EZE) can effectively lower the plasma LDL-cholesterol [7] and decrease the risk of CGD, as shown in the previous study [8]. In contrast to rodent NPC1L1, human NPC1L1 is highly expressed in the liver [9]. The hepatic function of NPC1L1 in mice promotes biliary cholesterol reabsorption and subsequently reduces biliary cholesterol concentrations [10]. Moreover, the function of hepatic NPC1L1 could be inhibited by EZE, as its glucuronide metabolite undergoes extensive enterohepatic recirculation [11], which causes the increase in biliary cholesterol saturation index [12, 13]. However, the lithogenic diet (LD) can cause whole-body lipid abnormalities [14]. Whether hepatic NPC1L1 can maintain the function of cholesterol transport under LD feeding remains unknown. Meanwhile, researchers discovered an increase in both hepatic and bile cholesterol accumulations in LDLR-deficient mice fed with LD and demonstrated that hepatic cholesterol remodeling also enhanced the risk of CGD in LD-fed mice [15]. Therefore, whether EZE can increase the risk of LD diet-mediated CGD progression by inhibiting hepatic NPC1L1 function remains subject to further investigation.

Fibroblast growth factor 15 (FGF15), the murine orthologue to human FGF19, is a critical regulator of bile acid biosynthesis and transport and affects the formation of cholesterol gallstones [16, 17]. Bile acid activates the nuclear receptor, farnesoid X receptor (FXR; NR1H4), and induces the expression of FGF15 [18]. Apart from decreasing CYP7A1, which limits cholesterol-like-bile acid conversion [17], FGF15 also decreases cholesterol absorption by inhibiting the expression of intestinal NPC1L1 [19]. However, no previous research has been conducted on the effect of FGF15/19 on the expression of hepatic NPC1L1.

This study revealed that NPC1L1^hepatic-OE^ mice fed a chow diet and a high fat cholesterol diet (HFCD), compared with WT mice, showed an increase in biliary cholesterol reabsorption, whereas mice fed with LD showed no difference. The LD feeding promoted hepatic NPC1L1 ubiquitination and subsequent degradation via activation of the FGF15-FGFR4 axis. EZE may act as a potent biliary cholesterol-desaturating agent in the development of CGD.

## Methods

### Creation of mNPC1L1^hepatic-OE^ mice, diet and drug treatment

All mice experiments were approved by the Animal Committee of Zhejiang University. Each experiment was conducted in triplicate. 3-month-old male C57BL/6 mice were purchased from the Model Animal Research Center of Nanjing University (Nanjing, China). All mice were raised in a pathogen-free animal facility under strict controlled conditions of temperature (23 °C), humidity (55 ± 5%) and lighting (12-hour light/dark cycle).

The cDNA sequences of murine NPC1L1 were obtained from the PubMed database (GenBank accession No. AY437866), synthesized by Shanghai GeneChem Co., Ltd. (Shanghai, China) and then cloned and packaged as Adeno-associated viruses (AAV) 2/8 [AAV2 inverted terminal repeat (ITR) DNA combined with the AAV8 capsid] virus with the thyroxine-binding globulin (TBG) promoter (which is a well-known and commonly used liver-specific promoter that permits the expression of exogenous genes with hepatocyte-specificity [20]). To study the effects of hepatic NPC1L1 gene overexpression with AAV2/8 gene delivery (AAV2/8-TBG-NPC1L1) on CGD progression, mice received an intravenous (via tail vein, i.v.) of AAV2/8-TBG-NPC1L1 or AAV2/8-TBG-Green fluorescent protein (GFP) at a dose of 1 × 10^11^ viral genomes (vg)/animal, AAV-TBG-GFP was used as control. Mice were fed a lithogenic diet (LD, containing 15.8% fat, 1.25% cholesterol, and 0.5% sodium cholate), a high-fat cholesterol diet (HFCD, containing 15.8% fat and 1.25% cholesterol), or a control chow diet (3% fat and 0.02% cholesterol) for 8 weeks, 5–10 mice for each group, from the Double Lion CO., LTD (Suzhou, China). WT mice were used as control. Mice were given diet and water ad libitum. The serum FGF15 levels were measured using the Enzyme-linked Immunosorbent Assay kit (LSBio LS-F11446; Seattle, WA). According to the instruction manual, we added 100 μL standard samples or serum collected from the portal vein of experimental mice [21] to coated plates and incubated for 2-hour at 37 °C. The results from each well were analyzed at 450 nm with an MD spectraMax i3x automatic microplate reader (Molecular Devices; San Francisco, CA). For drug treatment, ezetimibe (EZE, 5 mg/kg, oral dosing, once a day) was given 1 week prior to the beginning of the LD or HFCD. Ezetimibe is a drug with limited water solubility, thus the components of the HFCD or LD diet were thoroughly mixed with ezetimibe during diet preparation and the bacon-flavoured dough pills were produced by Double Lion CO., LTD (Suzhou，China) for the oral administration of ezetimibe [8]. The recombinant FGF15 (100 μg/kg/day, Abcam ab206457; Trumpington, UK) or highly selective covalent FGFR4 inhibitor H3B-6527 [22] (1702259–66-2, 5 mg/kg/day, once a day, i.p. injection; Sigma; Ronkonkoma, NY) were given during the start of the 7th week of feeding, respectively. The drug treatments were administered until the end of the experiments. The liver tissues, serum and bile samples were collected from euthanized mice and then snap frozen in liquid nitrogen and preserved at a temperature of − 80 °C until analysis.

### Immunoblot

The lysate proteins collected from experimental animals were lysed using complete protease inhibitor (Roche 11,697,498,001; Basel, Switzerland) and Thermo NE-PER™ nuclear and cytoplasmic extraction reagents (ThermoFisher 78,833, Waltham) on ice. The protein lysates collected from experimental mice and cells were separated with Sodium Dodecyl Sulfate Polyacrylamide Gel Electrophoresis and subsequently blotted onto nylon membranes to examine the binding of antibodies. The band densities of western blot images were analyzed with the ImageJ software. The following antibodies were used: NPC1L1 (ThermoFisher, PA1–16801), ABCB1 (ThermoFisher, MA1–26528), FGF15 (LSBio, LS-F11446), GFP (Abcam, ab291), tubulin (Abcam, ab7292) and ubiquitin (Abcam, ab134954). Other reagents included FGF15 (Abcam, ab206457), FGF19 (R&D, 969-FG), H3B6527 (Selleck, S8675), and cholic acid (Cayman, 20,250).

### Measurement of biliary lipid concentrations

The hepatic tissues (150 mg) from experimental mice were homogenized. The cholesterol (Wako 635–50,981; Osaka, Japan) and triglyceride (Wako 632–50,991; Osaka, Japan) contents in hepatic tissues and serum were examined by the DIAN Diagnostics Laboratory (Hangzhou, China). The cholesterol (Wako 635–50,981; Osaka, Japan), phospholipid (Wako 639–51,001; Osaka, Japan) and total bile acids (Alpha Laboratories 903,115; Hampshire, UK) contents in the bile were measured by the DIAN Diagnostics Laboratory (Hangzhou, China). The cholesterol saturation index (CSI) was calculated according to Carey’s critical table [23].

### Histological evaluation

The liver tissues from experimental mice were fixed in 4% formaldehyde, embedded in paraffin, sectioned at 5 μm, and then stained with hematoxylin-eosin (H&E, Beyotime C0105S; Suzhou, China), and Masson’s trichrome staining (Solarbio G1340, Beijing, China). The liver histology was assessed by two blinded independent observers who were blinded to the dietary condition. The amount of liver steatosis (percentage of lipid droplet-containing cells) was graded as 0 (< 5%), 1 (5–33%), 2 (> 33–66%) and 3 (> 66%). The stages of liver fibrosis were evaluated by measuring the degree of fibrosis. Stage 1: perisinusoidal fibrosis in liver zone 3; stage 2: perisinusoidal and portal fibrosis in liver zone 3; stage 3: perisinusoidal fibrosis, portal fibrosis, and bridging fibrosis; stage 4: cirrhosis. Images were obtained with DP70 camera coupled to IX71 microscope (Olympus; Tokyo, Japan).

### Cholesterol crystals examination

Gallbladder bile from mice was dispersed on glass slides and viewed using the Leica DM5000 polarized microscope.

### Quantitative reverse transcription polymerase chain reaction

All primer sequences utilized were listed in Supplemental Table 1. The expression of 18 s RNA was used to standardize the expression data.

### Cell culture and treatment

Human liver HepG2 cells (ATCC HB-8065, Manassas, VA) were plated in 24-well plates (5 × 10^4^ cells per well) and cultured in Eagle’s Minimum Essential Medium with 10% fetal bovine serum (ThermoFisher, Waltham, MA), incubated at 37 °C and 5% CO2. For drug treatment, cells were washed with 0.5 ml PBS and then switched to a fresh medium with drug, and incubated for 24 h. The following drugs were used in vitro: Recombinant FGF-19 (10 ng/mL ~ 20 ng/mL, 30-minute; R&D 969-FG; Minneapolis, MND); H3B-6527 (20 ng/mL, 30-minute; Sigma 1702259–66-2, Ronkonkoma, NY); Cholic acid (50 μM ~ 100 μM, 30-minute; Calbiochem CAS 361–09-1, San Diego, CA). To investigate the effect of the proteasome inhibitor MG132 (10 μM; Sigma M7449, Ronkonkoma, NY) on the ubiquitination state of NPC1L1, HepG2 cells were pre-treated with MG132 for 8-hour before FGF19 treatment.

### Statistics

The lipid concentrations in bile, serum and liver tissues, the bile CSI values and the mRNA expression of ABCG5, ABCG8, Cyp7a1, Cyp8b1, Cyp27a1 and Cyp7b1 in liver tissues were statistically compared between WT mice and mNPC1L1^hepatic-OE^ mice after 8-week feeding, with or without EZE, FGF15 or H3B6527 treatment. The statistical analysis was carried out with Prism 8.0. The normality of the data was determined using the Shapiro-Wilkes test. The Mann-Whitney U-test was used to compare data with non-normal distribution. The Student’s t-test was used to compare two independent groups with normal distributions. The *p* value less than 0.05 was considered significant.

## Results

### Hepatic NPC1L1 has no effect on CGD risk

**Fig. 1:**
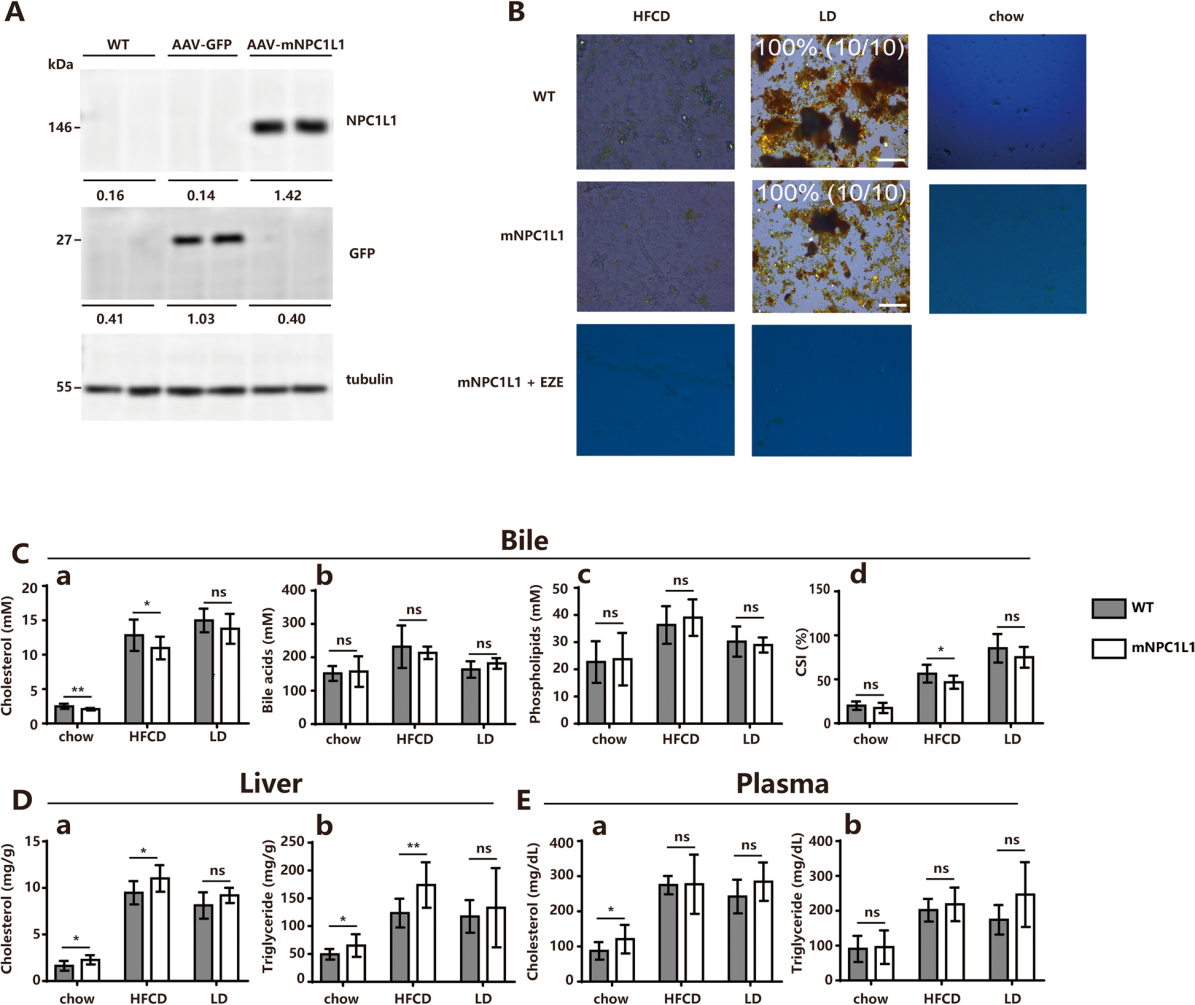
Hepatic NPC1L1 had no effect on bile cholesterol reabsorption in LD-fed mice. The WT and mNPC1L1^hepatic-OE^ mice were fed with 8-week chow diet, HFCD and LD with or without EZE treatment prior to the indicated experiments. **A** Immunoblotting analysis was applied to determine the hepatic expressions of GFP and NPC1L1 in chow diet-fed WT, AAV-GFP, and AAV-mNPC1L1 mice, with tubulin used as loading controls. The band densities of western blot images were analyzed with the ImageJ software and indicated below the bands, normalized to their loading controls. **B** Biliary cholesterol monohydrate crystals aggregating into gallstones and true gallstones examined by polarizing light microscopy. Original magnification, × 40. **C** Biliary cholesterol (a), bile acids (b), phospholipid (c) and (d) CSIs in a-d panels. **D** Cholesterol (a) and triglyceride (b) from liver tissues. **E** Cholesterol (a) and triglyceride (b) from plasma. Mann-Whitney U-test for data with non-normal distribution and Student’s t-test for normal distributions. *P < 0.05, **P < 0.01, ns no significance. WT, wide-type; EZE, ezetimibe; HFCD, high fat-cholesterol diet; LD, lithogenic diet; CSI, cholesterol saturation index

Mice expressing hepatic NPC1L1 (NPC1L1^hepatic-OE^ mice) were generated using Adeno-associated viruses (AAV) gene delivery (Fig. 1A). To investigate the effect of dietary components on hepatic NPC1L1 expression, HFCD was applied in this study, which is an essential dietary condition for inducing non-alcoholic fatty liver disease (NAFLD) [24] and differs from LD with only a 0.5% dietary cholic acid (CA) component. Gallstone formation rates showed no difference between WT and mNPC1L1^hepatic-OE^ mice fed with LD (Fig. 1B). When mNPC1L1^hepatic-OE^ mice were fed with a chow diet and HFCD, their biliary cholesterol concentrations were decreased, and their cholesterol and triglyceride levels in the liver were also considerably increased (Fig. 1C-E). Meanwhile, the cholesterol concentrations in bile and the liver were comparable between WT and mNPC1L1^hepatic-OE^ mice (Fig. 1C-E).

**Fig. 2:**
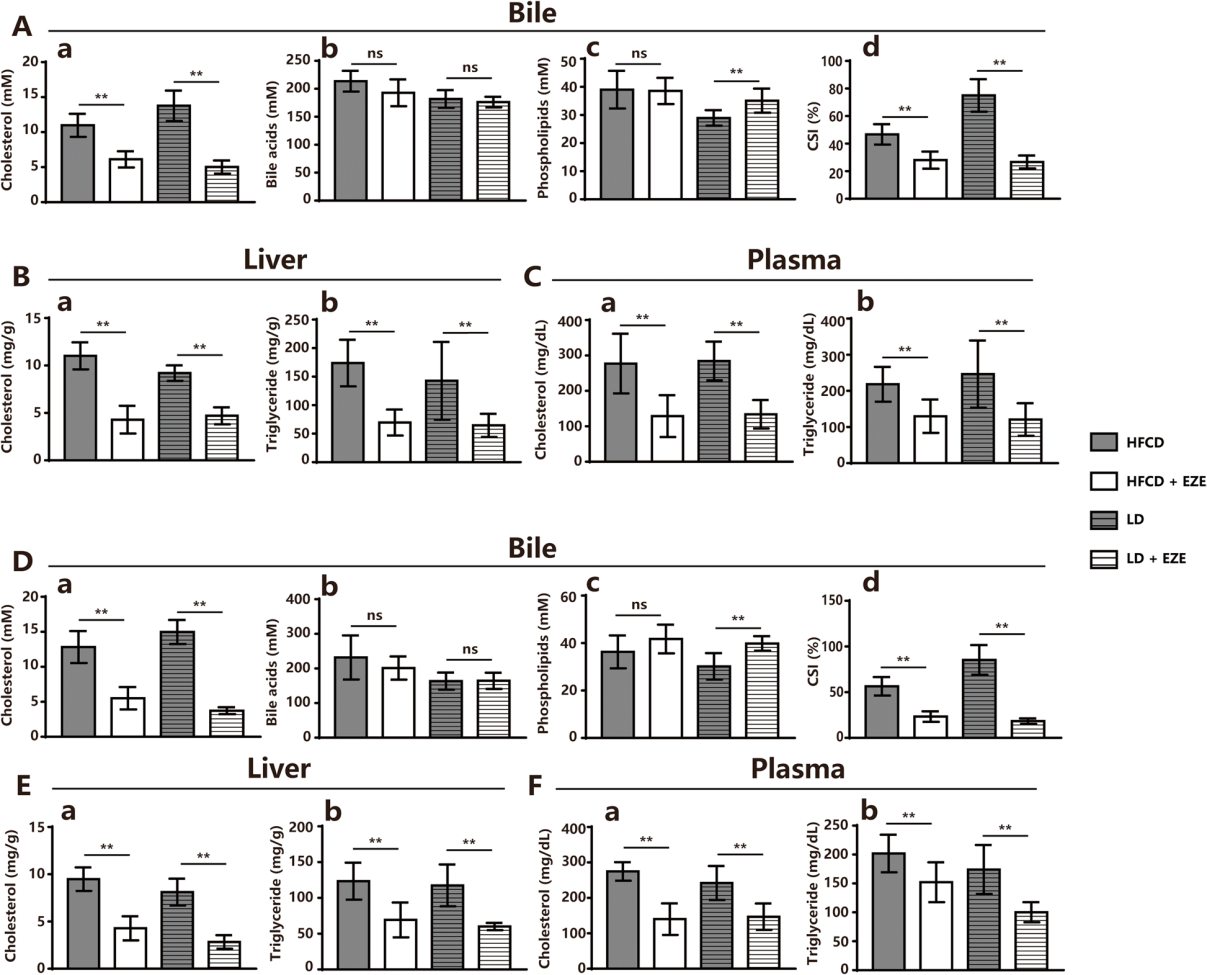
EZE treatment decreased bile cholesterol saturation in HFCD-fed mNPC1L1^hepatic-OE^ mice. The WT and mNPC1L1^hepatic-OE^ mice were fed with 8-week HFCD and LD, with or without EZE treatment prior to the indicated experiments. In mNPC1L1^hepatic-OE^ mice: **A** Biliary cholesterol (a), bile acids (b), phospholipid (c) and (d) CSIs in a-d panels. **B** Cholesterol (a) and triglyceride (b) from liver tissues. **C** Cholesterol (a) and triglyceride (b) from plasma. In WT mice: **D** Biliary cholesterol (a), bile acids (b), phospholipid (c) and (d) CSIs in a-d panels. **E** Cholesterol (a) and triglyceride (b) from liver tissues. **F** Cholesterol (a) and triglyceride (b) from plasma. Mann-Whitney U-test for data with non-normal distribution and Student’s t-test for normal distributions. *P < 0.05, **P < 0.01, ns no significance. EZE, ezetimibe; HFCD, high fat-cholesterol diet; CSI, cholesterol saturation index

Considering the inhibitory impact of EZE on hepatic NPC1L1, the possibility that EZE can increase the risk of CGD during HFCD feeding was further investigated. When treated with EZE, the HFCD-fed mNPC1L1^hepatic-OE^ mice exhibited a decrease in cholesterol concentrations in bile, the liver, and serum (Fig. 2A-C) with no gallstone formation (Fig. 1B), whereas the WT mice demonstrated a similar result (Fig. 2D-F). When the previous results were put together, it was found that hepatic NPC1L1 has no effect on the risk of CGD on HFCD and LD diets, while EZE can stop the intestinal absorption of cholesterol and prevent CGD.

### Hepatic NPC1L1 protein degradation in LD-fed mice

**Fig. 3:**
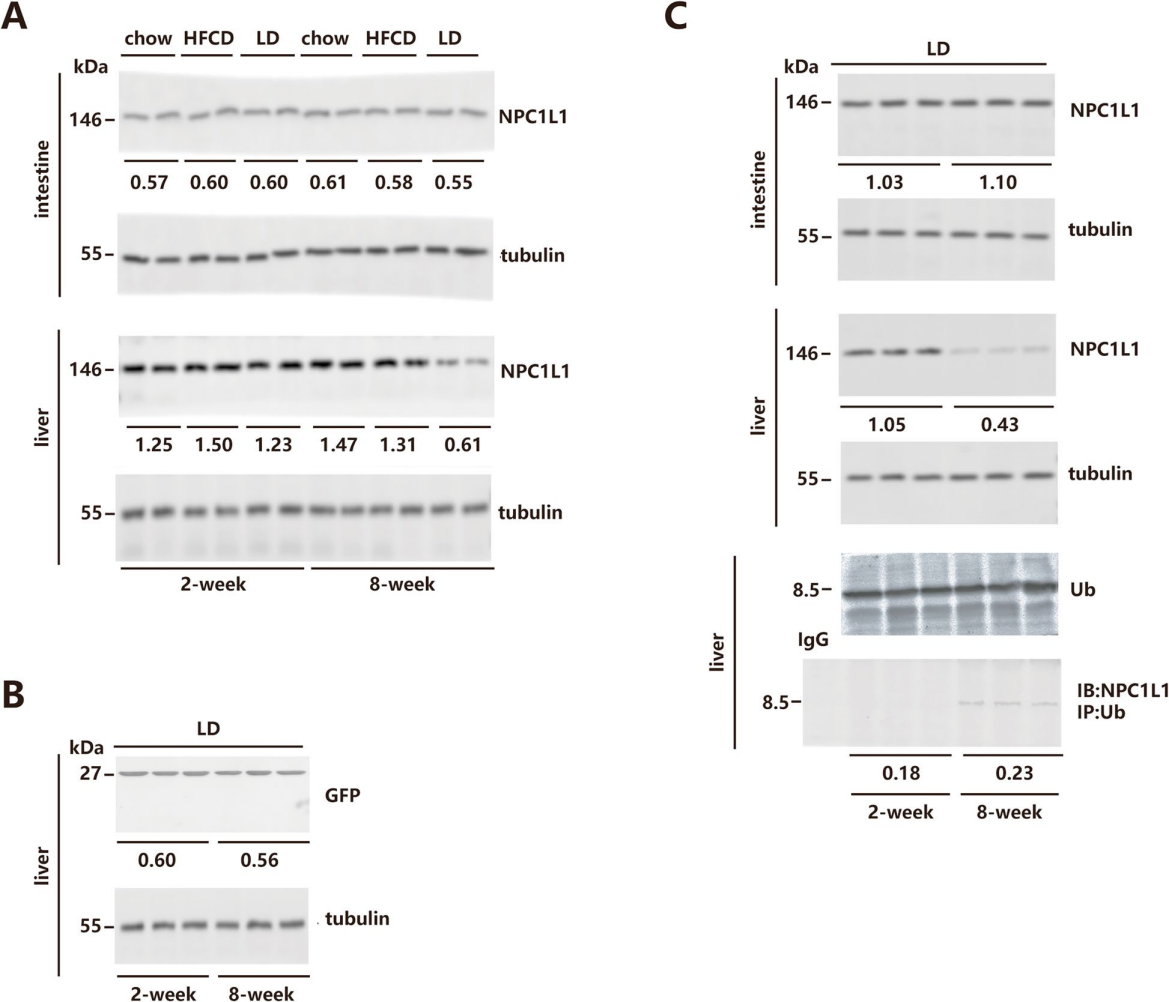
Hepatic NPC1L1 protein degradation in LD-fed mice. The mNPC1L1^hepatic-OE^ mice were fed with 2-week or 8-week chow diet, HFCD and LD prior to the indicated experiments. Immunoblotting analysis was applied to assess the expression of NPC1L1 in intestine and liver (**A**), and GFP in liver (**B**), with tubulin controls. **C** upper panel: Immunoblot of NPC1L1 and ubiquitin (Ub) in the liver collected from LD-fed mice. The antibody against tubulin served as the internal control. Lower panel: NPC1L1 ubiquitination was assessed by immunoprecipitating NPC1L1 and immunoblotting using anti-Ub antibody. The band densities of western blot images were analyzed with the ImageJ software and indicated below the bands, normalized to their loading controls. Student’s t-test. *n* = 3. HFCD, high fat-cholesterol diet; LD, lithogenic diet; Ub, ubiquitination

The hepatic and intestinal NPC1L1 expressions from mNPC1L1^hepatic-OE^ mice fed with a chow diet, HFCD, and LD were investigated. It was discovered that only the mNPC1L1^hepatic-OE^ mice fed with 8-week LD demonstrated a decrease in hepatic NPC1L1 expression, whereas intestinal NPC1L1 expression remained consistent across these diets (Fig. 3A). At the indicated time points following AAV-mNPC1L1 injection, hepatic GFP expression was comparable between 2- and 8-week LD-fed mice (Fig. 3B), suggesting that LD feeding did not affect the efficacy of long-term AAV-based gene delivery. Results subsequently revealed that ubiquitination and subsequent degradation of the hepatic NPC1L1 protein occurred in mNPC1L1^hepatic-OE^ mice after 8-week LD feeding (Fig. 3C).

### LD-induced FGF15-FGFR4 pathway promoted hepatic NPC1L1 protein turnover

**Fig. 4:**
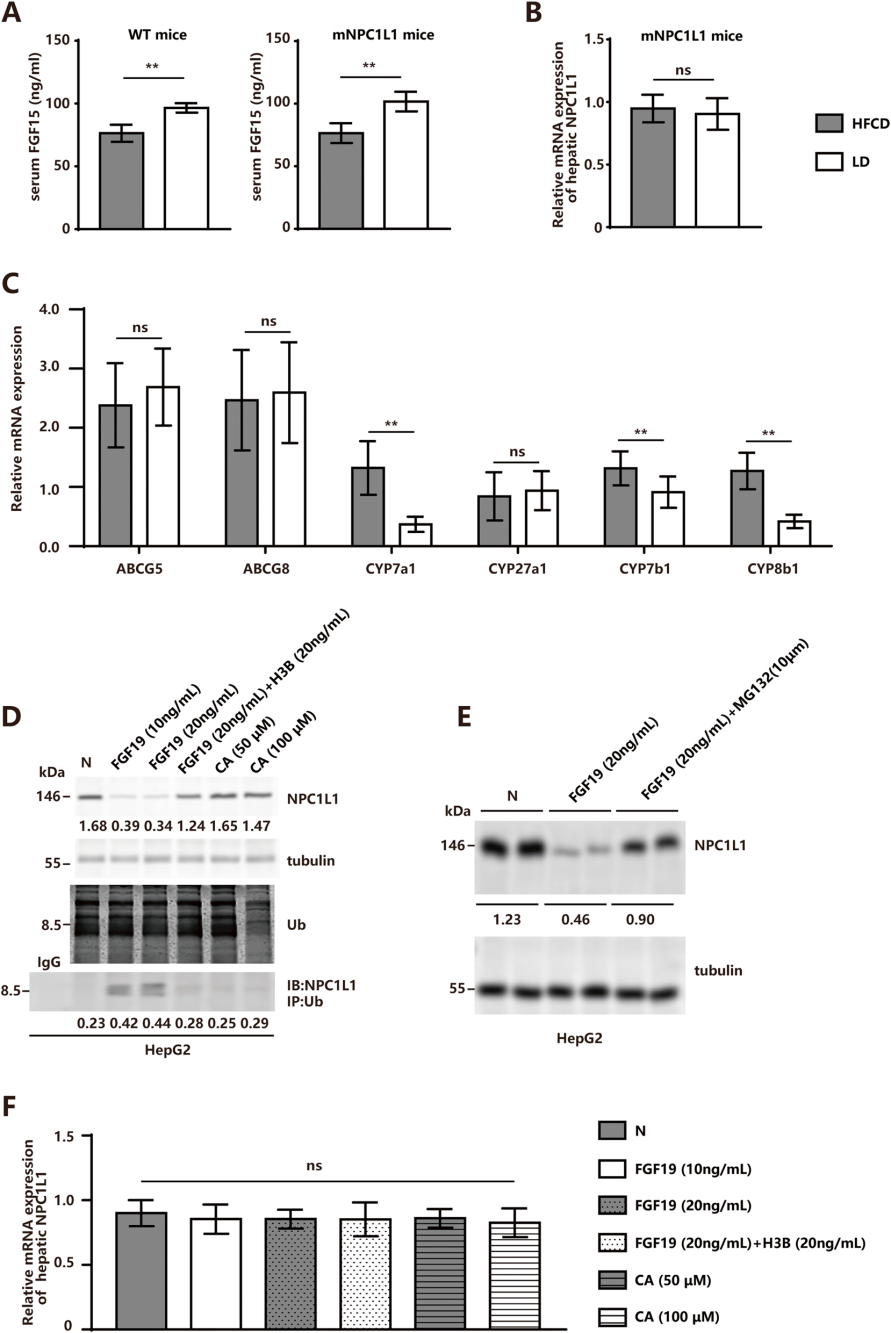
FGF15-FGFR4 pathway promoted hepatic NPC1L1 protein turnover. The WT and mNPC1L1^hepatic-OE^ mice were fed with 8-week HFCD and LD prior to the indicated experiments. **A** The serum FGF15 concentrations in WT and mNPC1L1^hepatic-OE^ mice. The hepatic mRNA expression of (**B**) NPC1L1, and (**C**) ABCG5/8, CYP7a1, CYP8b1, CYP27a1, CYP7b1 in mNPC1L1^hepatic-OE^ mice. **D** upper panel: Immunoblot of NPC1L1 and ubiquitin (Ub) from cultured human HepG2 hepatocarcinoma cells, treated with or without FGF19, H3B-6527 and CA. The antibody against tubulin served as the internal control. Lower panel: NPC1L1 ubiquitination was assessed by immunoprecipitating NPC1L1 and immunoblotting using anti-Ub antibody. **E** Immunoblot of NPC1L1 from cultured human HepG2 hepatocarcinoma cells, treated with or without FGF19 and MG132, with tubulin used as loading controls. The band densities of western blot images were analyzed with the ImageJ software and indicated below the bands, normalized to their loading controls. **F** The mRNA expression of NPC1L1 in cultured human HepG2 hepatocarcinoma cells, treated with or without FGF19, H3B-6527 and CA. Mann-Whitney U-test for data with non-normal distribution and Student’s t-test for normal distributions. *P < 0.05, **P < 0.01, ns no significance. CA, cholic acid; HFCD, high fat-cholesterol diet; LD, lithogenic diet; Ub, ubiquitination

LD contains a 0.5% CA component as compared with HFCD, which is required for the activation of the gut-liver cross-talk-initiated FGF15/19-FGFR4-SHP-Cyp7a1/8b1 pathway, which increases the risk of CGD by inhibiting the cholesterol-bile acid conversion. Compared to the HFCD-fed mNPC1L1^hepatic-OE^ mice, the LD-fed mNPC1L1^hepatic-OE^ mice showed increased serum FGF15 concentrations (Fig. 4A), whereas the hepatic NPC1L1 mRNA expressions were comparable (Fig. 4B). Meanwhile, the expressions of the bile acid synthesis enzymes CYP7a1 and CYP8b1 were decreased (Fig. 4C). With the use of in vitro cultured human HepG2 hepatocarcinoma cells, which expressed NPC1L1, it was shown that FGF19 instead of CA induced hepatic NPC1L1 protein ubiquitination, and the inhibition of FGFR4 (H3B-6527) rescued FGF19-induced hepatic NPC1L1 protein decay (Fig. 4D). When treated with MG-132, an inhibitor of the proteasome, the FGF19-treated HepG2 cells also demonstrated rescued hepatic NPC1L1 protein decay, confirming the ubiquitination of NPC1L1 protein ubiquitination induced by the FGF15/19-FGFR4 axis (Fig. 4E). The NPC1L1 mRNA expressions were comparable between HepG2 cells treated with or without FGF19, CA and H3B-6527 (Fig. 4F).

### FGF15-FGFR4 pathway decreased biliary cholesterol reabsorption through NPC1L1 protein turnover

**Fig. 5:**
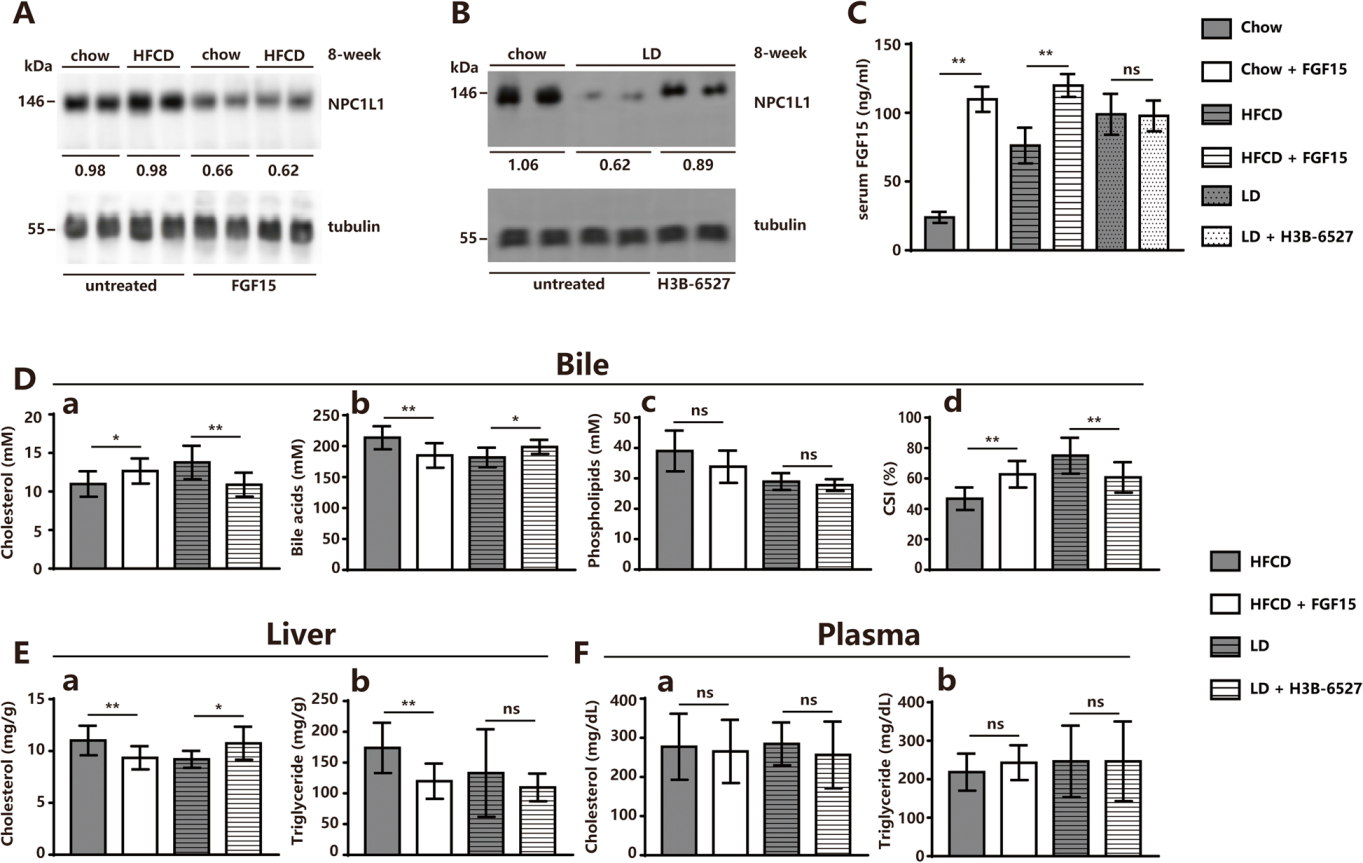
FGF15-FGFR4 pathway decreased biliary cholesterol reabsorption. Immunoblotting analysis was applied to assess the differences of hepatic NPC1L1 protein levels in mNPC1L1^hepatic-OE^ mice fed with 8-week chow diet and HFCD with or without FGF15 treatment (**A**), as well as those fed with 8-week chow diet and LD with or without H3B-6527 treatment (**B**), with tubulin controls. The band densities of western blot images were analyzed with the ImageJ software and indicated below the bands, normalized to their loading controls. **C** The serum FGF15 concentrations in mNPC1L1^hepatic-OE^ mice fed with 8-week chow diet and HFCD with or without FGF15 treatment, as well as those fed with 8-week chow diet and LD with or without H3B-6527 treatment. The (**D**) biliary cholesterol (a), bile acids (b), phospholipid (c) and (d) CSIs in a-d panels, **E** cholesterol (a) and triglyceride (b) from liver tissues, **F** cholesterol (a) and triglyceride (b) from plasma were from the mNPC1L1^hepatic-OE^ mice fed with 8-week HFCD with or without FGF15 treatment and mNPC1L1^hepatic-OE^ mice fed with 8-week LD with or without H3B-6527 treatment. Mann-Whitney U-test for data with non-normal distribution and Student’s t-test for normal distributions. *P < 0.05, **P < 0.01, ns no significance. HFCD, high fat-cholesterol diet; LD, lithogenic diet; CSI, cholesterol saturation index

**Fig. 6:**
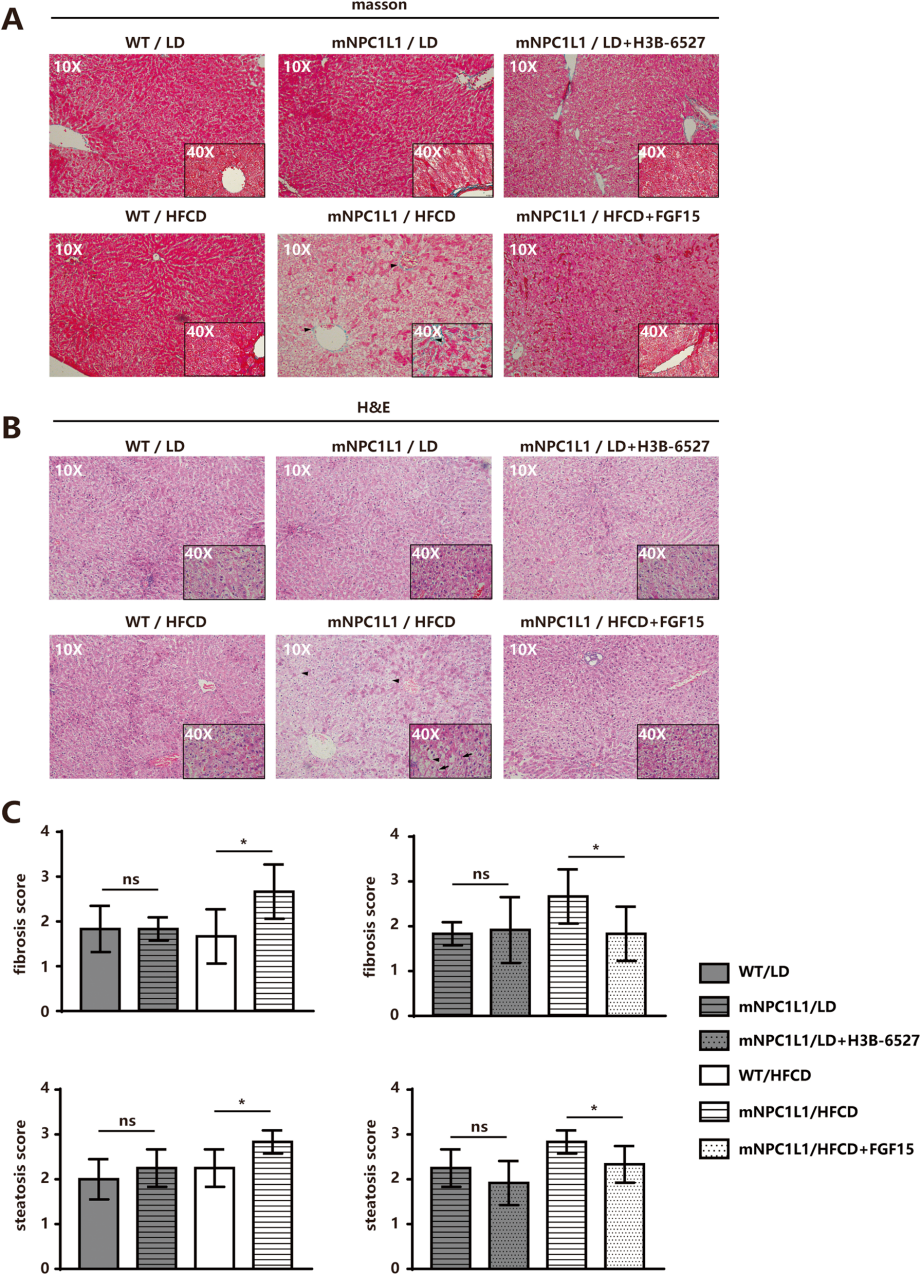
FGF15-FGFR4 pathway decreased liver cholesterol infiltration and fibrosis. The WT mice were fed with 8-week HFCD or LD. The mNPC1L1^hepatic-OE^ mice were fed with 8-week HFCD with or without FGF15 treatment, and LD with or without H3B-6527 treatment prior to the indicated experiments. **A** Masson staining of liver fibrosis in mice. The arrowhead pointed to the noticeable portion of perisinusoidal fibrosis, portal fibrosis, plus bridging fibrosis. Original magnification, × 10 and × 40. **B** HE staining of liver sections from mice. The arrowhead pointed to the noticeable portion of hepatocyte ballooning and the arrow pointed to the mallory-denk bodies. Original magnification, × 10 and × 40. **C** Fibrosis scores and steatosis scores in mice. *P < 0.05, **P < 0.01, ns no significance. HFCD, high fat-cholesterol diet; LD, lithogenic diet

**Fig. 7:**
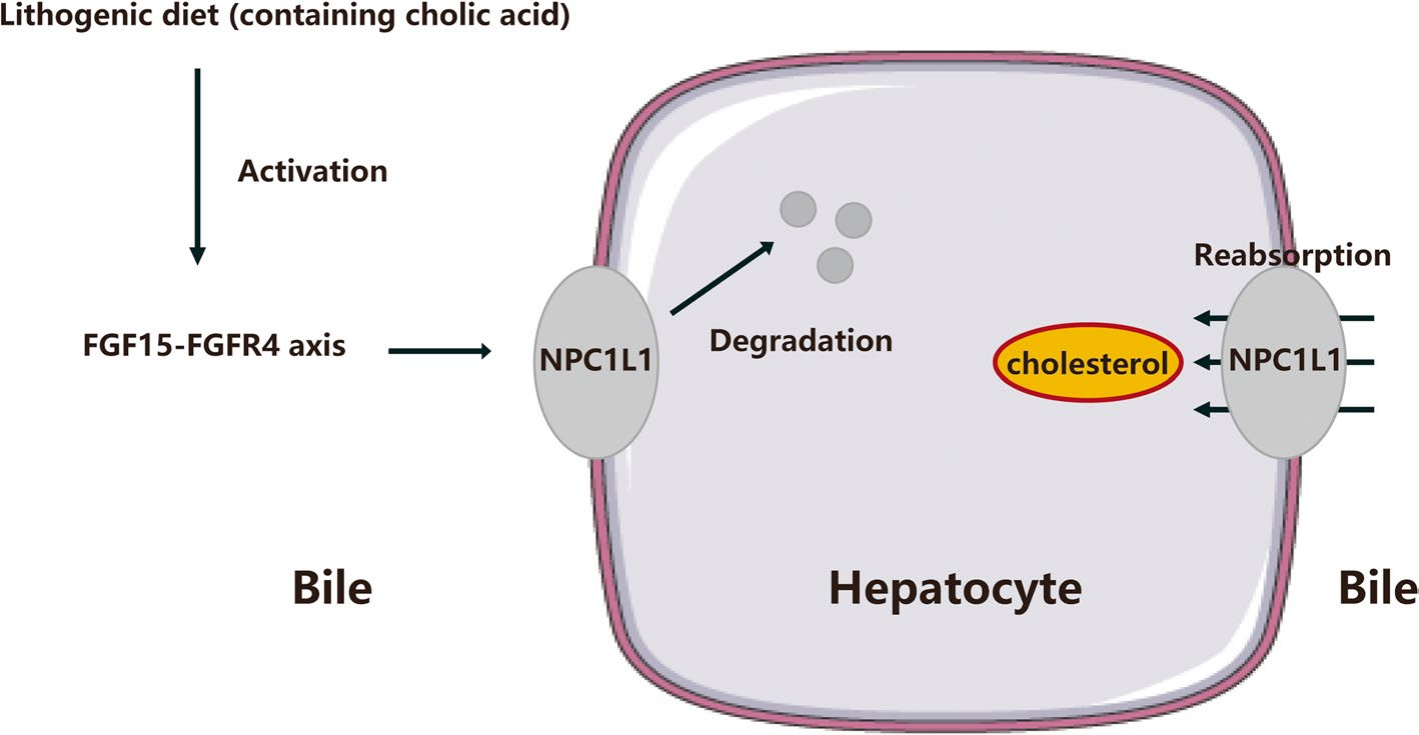
Role of hepatic NPC1L1 protein degradation in lithogenic diet-fed mice. The hepatic NPC1L1 protein located in plasma membrane can transport the cholesterol into hepatocytes from bile. When fed with LD, the cholic acid activated FGF15-FGFR4 axis would induce the ubiquitination and subsequent degradation of hepatic NPC1L1 in mNPC1L1^hepatic-OE^ mice. The hepatic NPC1L1 does not affect the cholesterol reabsorption and CGD development with LD feeding

The mediation of the FGF15-FGFR4 axis on hepatic NPC1L1 turnover in vivo was examined. The administration of purified FGF15 (400 mg per kg body weight, 7 days) during HFCD feeding stimulated hepatic NPC1L1 protein reduction, whereas the inhibition of FGFR4 (H3B-6527) (100 mg per kg body weight, 7 days) during LD feeding rescued FGF19-induced hepatic NPC1L1 protein decay in mNPC1L1^hepatic-OE^ mice (Fig. 5A-C). Meanwhile, the treatment of FGF15 during HFCD prevented bile cholesterol reabsorption, and H3B-6527 during LD also partially restored bile cholesterol reabsorption efficiency (Fig. 5D-F). When fed with HFCD, the mNPC1L1^hepatic-OE^ mice, compared with WT mice, showed increased lipid infiltration and fibrosis in the liver (Fig. 6). These findings implied that the high-bile-acid component in LD may destroy the cholesterol reabsorption activity of hepatic NPC1L1 via the FGF15-FGFR4 pathway (Fig. 7).

## Discussion

The importance of the cholesterol transporter NPC1L1 in the intestine for efficient absorption of cholesterol is well documented [4]. Elevated NPC1L1 expression raises the risk of CGD [13]. EZE treatment decreased cholesterol concentrations and cholesterol saturation indices in LD-fed WT mice, which could also inhibit the gallbladder stasis-induced cholesterol crystal nucleation [25–28]. However, humans highly express hepatic NPC1L1, and EZE may inhibit cholesterol reabsorption from bile and increase biliary cholesterol contents, thus potentially aggravating the risk of CGD [10, 29]. So, it’s still not clear whether EZE can be used safely to treat CGD in people.

This study evidenced that elevated FGF15 during LD feeding could induce hepatic NPC1L1 ubiquitination and subsequent degradation, which caused dysfunction of hepatic cholesterol reabsorption. Thus, EZE treatment could not promote cholesterol supersaturation through inhibiting hepatic NPC1L1, but retained the negative effect on intestinal NPC1L1 during LD feeding. This explained the previous results that EZE can prevent CGD in LD-fed NPC1L1^hepatic-OE^ mice. It was also found that HFCD-fed NPC1L1^hepatic-OE^ mice retained the function of hepatic NPC1L1 that could decrease biliary cholesterol supersaturation, which could be inhibited with EZE treatment. However, no CGD occurred in HFCD-fed NPC1L1^hepatic-OE^ mice when treated with EZE, and their biliary cholesterol concentrations were decreased. Considering that ezetimibe can inhibit intestinal NPC1L1 and thus reduce the cholesterol absorption in the gut-liver axis, the inhibition of NPC1L1 might have a relatively minor impact on the mediation of biliary cholesterol secretion. Meanwhile, in addition to biliary cholesterol supersaturation, the incidence of CGD is related to several factors such as reduced nucleation time and bacterial infection [30, 31]. In conclusion, the data can overcome the uncertainties about the potential side effect of EZE on hepatic NPC1L1 function, which is unfavorable to the prevention of CGD.

The differences in bile and liver cholesterol levels in 8-week HFCD-fed mice were statistically significant, which suggested that the AAV-mNPC1L1 treatment was effective without destroying of hepatic NPC1L1 function. Meanwhile, the stable expression of AAV-GFP in LD-fed mice also proved the validity of the AAV-mediated method. However, the expression levels and functions of hepatic NPC1L1 were considerably decreased at 8 weeks compared to 2 weeks in LD-fed AAV-mNPC1L1 mice but not in mice fed with HFCD or chow diet. The only difference between LD and HFCD was the addition of 0.5% dietary bile acid, which stimulates the FXR in the small intestine and promotes the synthesis of the FGF15 (FGF19 in humans) protein in the terminal small intestine [18, 32]. The FGF15-SHP axis decreases the intestinal NPC1L1 expression by suppressing the sterol regulatory element-binding transcription factor 2 [19], whereas the effect of the FGF15 on hepatic NPC1L1 remains unknown. In this study, the FGF15 treatment could induce the degradation of hepatic NPC1L1 both in vivo and in vitro. However, the intestinal NPC1L1 expression was unaffected by FGF15 treatment, which may due to the secreted FGF15 often acts as a paracrine/endocrine signal [33], and the FGF15 treatment was limited to 7 days in this study. Meanwhile, the FGF15-FGFR4 axis could regulate other cholesterol transport genes such as Acat2, a cholesterol esterification *trans*-acyltransferase, hence decreasing the concentration of cholesterol in bile [19, 34]. The activation of the FGF15-FGFR4 axis can inhibit the release of bile acid (BA) synthesis enzymes, which could explain why LD-fed NPC1L1^hepatic-OE^ mice, compared with HFCD-fed mice, demonstrated a decrease in CYP7a1, CYP7b1, and CYP8b1 [17]. Activating intestinal FXR signaling has been shown to decrease total cholesterol levels by raising hydrophilic bile acid levels, hence promoting transintestinal cholesterol excretion [35]. Furthermore, FGF19 stimulates gall bladder relaxation and bile filling following a meal [36]. Thus, the FGF15-FGFR axis exerts several effects on cholesterol gallstone disease. Meanwhile, the HFCD-induced gut microecology can lead to a decrease in T-β-MCA, which act as a natural inhibitor of endogenous FXR in mice [37]. Thus, the HFCD may promote, to some extent, the release of intestinal FXR-FGF15 signaling and the activation of hepatic FGFR4. LD feeding, on the other hand, can directly activate the gut-liver FGF15-FGFR4 axis via the components of the hydrophobic bile acid (endogenous FXR-FGF15 activator [38]), which occurrs earlier than HFCD. Given that hepatic NPC1L1 degradation was a long-term accumulation process that only occurred significantly in 8-week LD feeding, HFCD feeding may be insufficient to cause significant hepatic NPC1L1 protein degradation and complete hepatic NPC1L1 function elimination. In conclusion, this study discovered a novel mechanism for the FGF15-FGFR4 axis to contribute to bile cholesterol saturation during LD feeding by promoting hepatic NPC1L1 ubiquitination and subsequent degradation.

Through stimulation of the FGFR4-GS3Kβ-Nrf2 signaling pathway, FGF19 can protect cells against endoplasmic reticulum stress [39]. FGF19 therapy reversed the hepatosteatosis and ER stress that were exacerbated in HFCD-fed mice [40]. It has been evidenced that NPC1L1 protein was eventually degraded via the endoplasmic reticulum-associated degradation pathway, as the inactivation of valosin-containing protein (VCP) prevents NPC1L1 variants from degradation [41]. Since VCP plays a key role in ER stress and the endoplasmic reticulum-associated degradation pathway [42], future studies should look into whether FGF15 increases the ubiquitination and subsequent degradation of hepatic NPC1L1 by activating the VCP-UFD1-NPL4 complex.

Moreover, intestinal FGF15 expression is decreased in intestinal NPC1L1-deficient mice [43], and whether EZE therapy will inhibit the FGF15/19-FGFR4 axis and hence promote the risk of CGD remains to be verified. It was also shown that EZE treatment in dogs (expresses both hepatic and intestinal NPC1L1) increased the bile cholesterol concentrations [44]. Thus, the effect of EZE on CGD still requires further clinical trials, although the potential side effect of EZE on hepatic NPC1L1 during gallstone formation was excluded in this study.

### Strengths and limitations

This study has several strengths. It is the first to reveal the protein level and function of hepatic NPC1L1 in mice fed a long-term (8-week) diet. The generation of the NPC1L1^hepatic-OE^ mouse model and long-term dietary feeding can better simulate the process of CGD formation in humans. It is also reveal that hepatic NPC1L1 protein cannot maintain stable presence and cholesterol transport function during LD feeding because of the activation of the FGF15-FGFR4 axis, which therefore cannot affect CGD development.

It should be acknowledged that this study had limitations. In this work, the NPC1L1^hepatic-OE^ mice were driven by the AAV infection method, and we used either exogenous FGF15 or FGFR4 inhibitor H3B-6527 to study the role of the FGF15-FGFR4 axis on hepatic NPC1L1 protein stability in the short term. Next, we want to generate hepatic NPC1L1 transgenic mice to explore the impact of the FGF15-FGFR4 axis on hepatic NPC1L1 protein ubiquitination and its function during an 8-week CGD development, utilizing AAV-directed changes in the gut-liver FGF15-FGFR4 signaling. Meanwhile, it was found that hepatic NPC1L1 was significantly damaged and ceased to function as a cholesterol transporter during LD feeding. When compared to mice, humans have more hydrophobic bile acid profiles, which means that the function of human hepatic NPC1L1 needs more research.

## Conclusion

In conclusion, the activation of the FGF15-FGFR4 pathway in vivo during lithogenic diet feeding promoted the ubiquitination and degradation of hepatic NPC1L1. This study indicated that hepatic NPC1L1 cannot prevent CGD. These results demonstrate that ezetimibe can be an effective cholesterol absorption inhibitor for the therapeutic prevention of CGD in clinical trials.

## Supplementary Information

**Additional file 1.**

## Data Availability

Data is available upon request.

## Abbreviations

CGD: Cholesterol gallstone disease
NPC1L1: Niemann-Pick C1-like 1
EZE: Ezetimibe
WT: Wide-type
HFCD: High fat-cholesterol diet
LD: Lithogenic diet
CSI: Cholesterol saturation index
Ub: Ubiquitination
CA: Cholic acid
