# Supplementary material for: FGF15 promotes hepatic NPC1L1 degradation in lithogenic diet-fed mice
Source: Lipids Health Dis. 2022 Oct 8;21:97. doi: 10.1186/s12944-022-01709-8 (PMC9547418; doi:10.1186/s12944-022-01709-8)
Supplement: Supplementary file 1 — Additional file 1. [file 12944_2022_1709_MOESM1_ESM.docx]

| **Primers used for qRT-PCR** | | |  |
| --- | --- | --- | --- |
| gene symbol (mice) | forward primer (5' → 3') | reverse primer (5' → 3') |  |
| 18s RNA | GATGGGAAGTACAGCCAGGT | TTTCTTCAGCCTCTCCAGGT |  |
| ABCG5 | AGAGTCAGGATGGCCTGTAT | ATGCTGAGCAGGGCCACTAT |  |
| ABCG8 | GAGAGCTTCACAGCCCACAA | GCCTGAAGATGTCAGAGCGA |  |
| Cyp27b1 | GCCTCACCTATGGGATCTTCA | TCAAAGCCTGACGCAGATG |  |
| Cyp7a1 | TACAGAGTGCTGGCCAAGAG | TTCAAGGATGCACTGGAGAG |  |
| Cyp7b1 | AATTGGACAGCTTGGTCTGCCT | TGTGTATGAGTGGAGGAAAGAGGG |  |
| Cyp8b1 | GGCTGGCTTCCTGAGCTTATT | ACTTCCTGAACAGCTCATCGG |  |
| NPC1L1 | GAGGCCATCTGCTTCTTTCTAG | GTCAAAGATGATTGCTAAGCCA |  |
